# First-episode psychosis in a 15 year-old female with clinical presentation of anti-NMDA receptor encephalitis: a case report and review of the literature

**DOI:** 10.1186/s13104-016-2180-6

**Published:** 2016-07-29

**Authors:** Maria Moura, Amilcar Silva-dos-Santos, Joana Afonso, Miguel Talina

**Affiliations:** 1 Unit of Child and Adolescent Psychiatry, Department of Psychiatry, Hospital Vila Franca de Xira, R. do Parque Res. dos Povos 1, 2600-009 Vila Franca de Xira, Portugal; 2 Department of Psychiatry, Hospital Vila Franca de Xira, R. do Parque Res. dos Povos 1, 2600-009 Vila Franca de Xira, Portugal; 3 Faculty of Medicine, Institute of Pharmacology and Neurosciences, University of Lisbon, Lisbon, Portugal; 4 Unit of Neurosciences, Institute of Molecular Medicine, University of Lisbon, Av. Professor Egas Moniz, 1649-028 Lisbon, Portugal; 5 Child and Adolescent Psychiatry Unit, Hospital Dona Estefânia–Centro Hospitalar de Lisboa Central, R. Jacinta Marto, 1169-045 Lisbon, Portugal; 6 CEDOC, Chronic Diseases Research Centre, Nova Medical School, Faculdade de Ciencias Médicas, New University of Lisbon, Campo Mártires da Pátria 130, 1169-056 Lisbon, Portugal

**Keywords:** Anti-NMDA receptor encephalitis, First-episode psychosis, Adolescent, Psychosis, Case report

## Abstract

**Background:**

Anti-NMDA receptor encephalitis is an autoimmune disease that was identified in 2007, and manifests in a stepwise manner with psychiatric, neurological and autonomic symptoms. The disease is caused by autoantibodies against NMDA receptors. It can have a paraneoplastic origin, mainly secondary to ovarian teratomas, but it can also be unrelated to the tumor. This disease can affect both sexes and all ages.

**Case presentation:**

Here, we present a case of a 15 year-old female adolescent with first-episode psychosis with anti-NMDA receptor encephalitis not related to tumor, which manifested with delusion, hallucinations, panic attacks, agitation, and neurological symptoms, and later with autonomic instability. She was treated with immunotherapy and psychiatric medication resulting in improvement of her main psychiatric and neurological symptoms.

**Conclusion:**

Our main objective in presenting this case is to alert clinicians to this challenging and recent disease that has a clinical presentation that might resemble a functional psychiatric condition and can be underdiagnosed in the context of child and adolescent psychiatry.

## Background

Anti *N*-methyl-d-aspartate (NMDA) receptor encephalitis is an autoimmune disease that was initially identified as paraneoplastic syndrome in young women with ovarian teratomas [1]. Although some case reports of encephalitis in patients with ovarian teratoma had been published since 1997 [2–6], a definitive association between anti-NMDA receptor antibodies and encephalitis was established by Josep Dalmau and colleagues in 2007 [7]. Since then, several case reports, case series, multicenter studies and other works have been published. In addition to medical literature, this disease has become known by lay media and the general population. In 2012 the New York Post writer Susannah Cahalan published an autobiographical book titled *Brain on Fire*, where she described her experience with anti-NMDA receptor encephalitis that was dominated by presentation of delusion, hallucinations, and later with neurological symptoms [8]. The clinical presentation of anti-NMDA receptors encephalitis includes three main stages: (1) an initial period with viral prodrome and common cold-like symptoms that can last up to 1 week; (2) an intermediate stage, that can last from 1 to 3 weeks, mainly with psychiatric symptoms such as delusions, hallucinations, mania, agitation, changes in speech and disorganization (often seizures); and (3) prominent neurological symptoms, such as movement abnormalities, dysautonomia, hypoventilation and seizures that can last from weeks to months, with possible need for intensive care unit support [9]. Anti-NMDA receptor encephalitis affects both sexes and has been observed in all ages, although it is more frequent in young adults and children with or without teratomas [10, 11]. Exact prevalence of this disease is unknown, but according to epidemiological studies it is the most common cause of autoimmune encephalitis, ranking immediately after acute demyelinating encephalomyelitis [12]. According to the California Encephalitis Project, anti-NMDA receptor encephalitis is more frequent than any specific viral encephalitis [13]. This disease is fatal if left untreated, but patients improve with adequate measures, including intensive care support, immunotherapy and prolonged hospital stay with multidisciplinary care [10, 14]. When there is an underlying tumor, such us an ovarian teratoma, the prognosis after tumor resection is usually better than in the absence of tumor [10]. Recovery is usually slow; can take years, and may be associated with prolonged neuropsychiatric deficits [15]. Usually, psychiatrists are the first physicians to observe these patients because of the prominent presentation of behavioral symptoms, mood changes, and psychotic symptoms [10].

Here, we present a case report of a 15 year-old teenager with presentation mentioned above, where the diagnosis of anti-NMDA receptor encephalitis was considered and confirmed by detection of anti-NMDA antibodies in spinal fluid and clinical improvement after immunotherapy. Time between onset of symptoms and diagnosis was 23 days, which illustrates the difficulties in establishing diagnosis of anti-NMDA receptor encephalitis (a pleomorphic condition and a relatively new disease), which is why clinicians are not well aware of it. We highlight the importance of considering timely diagnosis, since it has a specific treatment with dramatic effect in the prognosis of these patients.

## Case presentation

A previously healthy 15 year-old female presented with behavioral changes with incoherent speech, restlessness, anxiety, and expressed the feeling that “everything is happening to me” after attending a 2-day spiritual retreat. One week before the presentation of psychiatric symptoms, she had unspecific common cold symptoms, but with no fever. On the second day of psychiatric presentation, she had a transient period of agitation and visual hallucination with spontaneous remission (she saw Jesus and her deceased grandparents). On the third day, she was brought to the emergency room (ER), for the first time, owing to an episode of agitation and speech blockade that happened at school. Concomitantly, she had somatic manifestations, such as swallowing impairment, odynophagia for water and food refusal. She showed regressive behavior, such as asking for help for basic hygiene and refusal to sleep alone. At this point, episodic speech changes, such as echolalia and verbal perseveration and intermediary insomnia were documented. Of note, there were no changes in the flow of thoughts or indication of disorganized behavior. The patient was medicated with risperidone (0.5 mg once a day) by the ER child and adolescent psychiatry attendant, and referred to the Child and Adolescent Psychiatry Outpatient Clinic. However, a few hours later, she was re-admitted in the pediatric ER with panic symptoms (tachypnea, tachycardia, shaking hands, and sweating). She was discharged and medicated with lorazepam (0.5 mg two times a day) and risperidone (1 mg once a day) with no improvement, namely, the visual hallucination persisted.

At the first child and adolescent psychiatry outpatient appointment (day 7 after symptoms onset), she was oriented, and presented with unexpressive facial mimicry, provoked speech, and psychomotor retardation. No changes in the form, rhythm or flow of thoughts, or delusions were documented. The patient had anxiety with no mood changes, although she had a regressive attitude. The patient was medicated with anxiolytics (diazepam 5 mg two times a day), and risperidone (1 mg) was increased to twice daily. At the next child and adolescent psychiatry appointment (day 10 of the disease), she maintained episodes of agitation with tremor of the upper limbs, screaming, and a desperation gaze that was attributed to hallucinations, according to her family. During these episodes, the patient said that “they are after me (…) I am a saint (…) nobody will survive…”. On other occasions she would experience periods of elation, with tendency to sing and express grandiose delusions: “I will be the best student at my class”. During the mental state examination she was extremely sleepy, calm with provoked speech, and answered with monosyllables. Therapy was adjusted, and risperidone was replaced with olanzapine (10 mg once a day). She was recommended for hospital admission, but the patient’s family refused this recommendation.

On day 18 of the disease, with no signs of clinical improvement, the patient was brought to a new appointment, and admitted in a psychiatry ward. At admission she was oriented to time, place, person and situation but showed periods of negativism, mutism, echolalia, echopraxia, motor agitation, upper limb tremor (that occurred at rest, with sudden onset and remission and disappeared when she was distracted), These symptoms occurred in bursts alternated with periods of normal speech but with pervasive prostration. Also, she was unable to take care of her own hygiene. At physical examination she did not have fever or changes in blood pressure, and cardiac or respiratory frequency. Pulmonary and cardiac auscultation were unremarkable, abdomen examination was also normal, and at the examination she had no changes of the skin and upper and lower limbs. At that point, the following diagnostic hypotheses were considered: prodromal phase of an affective psychosis, anxiety disorder with conversion traits, dissociative disorder (depersonalization disorder), organic disorder, and psychosis with catatonic features. Due to the catatonic symptoms, the diagnosis of neuroleptic malignant syndrome was also raised and a possible treatment with dopamine agonist was considered. However, this specific intervention was deferred since the patient had no fever or changes in the blood pressure, cardiac or respiratory frequency or laboratory imbalance. The diagnostic hypotheses were based on the clinical presentation, physical examination, and mental state examination. We did not apply the mini-mental state examination or any other rating scale for psychosis, mood, cognitive or neurologic symptoms.

Laboratory results and brain CT scan were normal. Psychiatric medication for psychosis was started. At day 5 after admission (day 23 of clinical disease), a lumbar puncture was performed with screening for antibodies for anti-NMDA receptors in the spinal fluid. Since the hypothesis of anti-NMDA receptors encephalitis was raised, it is mandatory to first rule out possible underlying neoplasm. Because the patient was female and ovarian teratoma can occur in up to 59 % of cases [10], a pelvic ultrasound was performed, but no changes were found. On the same day, the patient experienced tachycardia and decreased consciousness, with the need for intensive care unit (ICU) admission. No rating scale was applied during the stay in the intensive care unit. However, antibodies for NMDA receptors were detected and diagnosis of anti-NMDA receptor encephalitis was established.

At the ICU the patient experienced periods of delusion and agitation with loud screaming. She was medicated with midazolam (2 mg intravenous if needed), oral lorazepam (1 mg three times a day), and chloral hydrate (1 g every 6 h). Within the first few hours, risperidone was introduced at 0.5 mg daily and then titrated to 1 mg daily. She was subsequently treated with immunoglobulin (2 mg/kg/day), methylprednisolone (30 mg/kg/day), and plasmapheresis and rituximab (375 mg/m^2^/week). Regarding the maintenance psychopharmacology, the patient was on risperidone (1 mg two times a day) and lorazepam (2.5 mg if needed).

Two months after specific treatment for anti-NMDA receptor encephalitis, the patient still had difficulty with verbal articulation, but there were no changes regarding form, course, rhythm or thought content, and her mood was euthymic. She reported periods of emotional lability, although less frequent than before treatment, and there were no auditory or visual hallucinations. She reported moderate verbal memory impairment, attention and concentration difficulties at school. Although a formal neuropsychological evaluation was not performed, it was known that before disease onset, the patient had no cognitive difficulties at school since her IQ was considered within the normal range, and her grades were average. She started psychotherapy, physiotherapy, and learning support at school. She had progressive clinical improvement but still experienced emotional lability, anxiety, and verbal memory impairment, attention and concentration difficulties at school that justified her enrolment in a vocational educational school. Ten months later she had a seizure episode and a new treatment cycle was started. Since she experienced depressive symptoms after the encephalitis episode, sertraline (25 mg once a day) was added to her medication regimen. As of this writing, she is medicated with oxcarbazepine (450 mg two times a day), quetiapine SR (100 mg at bedtime), sertraline (25 mg once a day) and lorazepam (1 mg if needed). Also as of this writing (almost 3 year after the disease onset), the patient’s psychiatric symptoms have improved, but she still experiences verbal memory impairment, and attention and concentration difficulties.

## Discussion

In this present case, it was clear that we were not facing a common psychiatric condition. A diagnosis of an organic psychiatric disorder was considered because of the presence of atypical psychiatric symptoms such as visual hallucination and also by the uncommon sequence and mixture of psychiatric and neurologic symptoms. A diagnosis of anti-NMDA receptor encephalitis was hypothesized because of the following course of the illness: 1 week before symptoms onset the patient experienced common cold-like symptoms; day 1 of symptoms onset, she experienced incoherent speech, restlessness and expressed the feeling that “something was happening”; day 2, she presented with visual hallucinations and agitation; day 3, she was agitated, had speech blockade, had difficulty swallowing and refused to eat; day 5, she displayed panic attack; day 8, she experienced psychomotor retardation, speech changes, and regressive posture; day 10, she displayed agitation, tremor in her upper limbs, mood elation, and persecutory and grandiose delusions; day 18, she had episodes of negativism, mutism, tremor, echopraxia, prostration, and neglected her hygiene. At that moment she was admitted into a psychiatry ward. Blood workup and brain CT scan were normal. At day 23 of the disease (5 days after her admission in the psychiatry ward), a lumbar puncture was performed to test for auto-antibodies against NMDA receptors, and owing to decreased consciousness and catatonia she was admitted to an ICU. This clinical picture is consistent with the several cases of anti-NMDA receptor encephalitis that have been reported in literature [10, 15–21]. Anti-NMDA receptor encephalitis is an autoimmune disease that was first identified as a paraneoplastic syndrome in young women with ovarian teratomas, and can also occur as a rare manifestation of teratoma of the mediastinum, or due to testicular cancer or small-cell lung carcinoma [7, 10]; however it can also be non-paraneoplastic [3, 22]. The clinical presentation usually consists of three stages: a prodromal period that lasts up to 1 week with common cold-like symptoms, fever, headache, malaise or gastroenteritis; an intermediate period, lasting 1–3 weeks, with behavioral changes, psychiatric symptoms or mood changes. The most common clinical presentation resembles acute psychosis (anxiety, agitation, paranoia, auditory or visual hallucinations) [7, 23], meaning that psychiatrists are usually the first physicians to observe these patients. The third stage can last from weeks to months, and consist of neurological and autonomic symptoms, including alteration in speech, catatonia, and seizures. The common autonomic symptoms include urinary incontinence or sleep dysfunction. The more severe autonomic symptoms, such as hypoventilation, dysrhythmia, tachycardia, and hyperthermia, are not frequent in children [11]. It has been shown that this condition is more frequent in children than was previously thought and that its clinical manifestations in the pediatric population are similar to those of adults [11].

The exact pathophysiology of anti-NMDA receptor encephalitis is not fully understood. It is believed that NR1 subunits expressed by tumoral neural tissues may break the immune tolerance [10]. However, anti-NMDA receptor encephalitis is not always related to teratoma or other malignancies, and hence, other immunologic mechanisms might be involved. Hypotheses thus far proposed include the concept that there is an immunological response to a flu-like prodromal phase possibly related to a viral infection, and genetic susceptibility [10]. It is well known that NMDA plays an important role in the mechanism of psychosis, which is supported by association studies on hypofunction of glutamate and psychosis symptoms [24]. Also, immunoglobulin against NR1 can induce a reversible hypofunction of the glutamate receptors by triggering internalization of the receptors [25]. In our patient, the underlying etiology was unknown, but we cannot rule out a possible viral mechanism, since she had a short period of common cold-like symptoms before symptoms onset, and no tumor was detected.

Regarding the diagnosis, brain imaging is not helpful because there are no specific imaging biomarkers associated with this syndrome, though most patients may show minor nonspecific abnormalities during brain imaging [10]. For example, an MRI may show cerebral, cerebellar or brainstem hyperintensities [10]. Transient contrast enhancements of the cerebral cortex, meninges, and basal ganglia have also been noted [10]. Furthermore, though not specific, EEG may show epileptiform activity or diffuse slow waves [10]. The cerebrospinal fluid may have pleocytosis, high protein levels, and oligoclonal bands. Diagnosis is made on the presence of anti-NMDA receptor antibodies in the spinal fluid and/or in the blood [10]. Underlying neoplasm should be ruled. Since ovarian teratomas are the most frequent tumors associated with anti-NMDA receptor encephalitis, at least an ultrasound or MRI of the abdomen and pelvis must be considered in the initial diagnosis, and periodically for at least 2 years after the episode. However, attention should be paid to other tumors such as the more frequently associated with this autoimmune disorder (e.g. extraovarian teratomas, testicular, lung and breast tumors, ovarian carcinoma, thymic carcinoma or pancreatic cancer) [15]. Since virtually all tumor may trigger the production of autoantibodies against NMDA receptors, clinicians should consider the assessment of a possible underlying tumor according to the signs and symptoms of each clinical case. In our patient, the blood chemistry was normal and she had no alterations on brain CT. Since she was a 15 year-old adolescent, a pelvic ultrasound was performed to rule out tumor or teratomas, but no changes were found. Lumbar puncture was performed 5 days after her admission (23 days after disease onset) and was found to be positive for anti-NMDA receptor auto-antibody. At that time the patient also experienced autonomic instability, namely tachycardia, and also more severe neurological symptoms, such as decreased consciousness that justified her admission to the ICU. These findings in our patient were also consistent with the third stage of the course of the disease that can culminate in admission to the ICU. We further highlight the fact that diagnosis was made 23 days after symptoms onset. The delay was mainly due to the fact that the disease was reported for the first time only 10 years ago as of this report, which means that the clinicians were not yet aware of this syndrome. Since the course of the disease usually presents with psychiatric symptoms, and adult psychiatrists or child psychiatrists are the first physicians to see the patients, usually a preliminary diagnosis of a functional psychosis is made and psychopharmacology treatment is started. Indeed, a similar scenario happened with our patient, since she was admitted to the psychiatric ward after failure to respond to antipsychotics and benzodiazepines. However, we emphasize the fact that the pleomorphic presentation of the disease raised the hypothesis of possible atypical psychiatric conditions.

In terms of treatment for anti-NMDA receptor encephalitis, if a neoplasm is detected, tumor removal and immunotherapy (corticosteroids, intravenous immunoglobulin or plasma exchange) are the first-line treatments. In such cases, second-line immunotherapy (cyclophosphamide and/or rituximab) is seldom needed. However, in cases unrelated to tumors, first-line therapy tends to be less efficient, and escalation to second-line immunotherapy is frequently necessary [7]. Significant improvement occurs in more than two-third of patients after 2–3 months of therapy, which correlates with decreasing antibody titers. Symptoms resolution is inversely proportional to the order of symptom development [7]. Our patient was treated with immunoglobulin (2 mg/kg/day) and methylprednisolone (30 mg/kg/day), plasmapheresis, rituximab (375 mg/m^2^/week) and risperidone (1 mg two times a day) and lorazepam (2.5 mg if needed). Since she had no tumor, her prognosis was worse than if she had a neoplasm. Relapses have been documented in literature [10]. Ten months after symptoms onset, our patient had a seizure episode and a new treatment cycle was started. These findings are also consistent with the literature since the long course of the disease is characterized by slow recovery that can take years, and may be associated with prolonged neuropsychiatric deficits [15]. Presently (almost 3 years after the disease onset), the psychiatric symptoms have improved, but she still experiences verbal memory impairment, attention and concentration difficulties. According to the literature, long-lasting cognitive deficits, mainly memory impairment and executive dysfunction may occur in about 88.8 % of the patients [26]. Although a recent study (using MRI of patients after anti-NMDA receptor encephalitis), reported that hippocampal volumetric and microstructural impairments correlate with memory performance [27], in our patient, we did not perform any further imaging study such as MRI.

Regarding the outcome, the main good prognostic factors are: early detection and intervention, presence and subsequent removal of an underlying tumor, good response to first line immunotherapy, no need for intensive care unit admission, and the absence of relapses [10, 15]. Our patient had more bad than good prognostic factors since she had no tumor, the diagnostic was made 23 days after symptoms onset, she experienced autonomic symptoms, she was subsequently admitted to an intensive care unit and had a relapse 10 months after the symptoms onset.

Finally, we would like to highlight that even though anti-NMDA receptor encephalitis is classically described as having a step-wise presentation with a prodromal flu-like phase followed by periods of psychiatric, neurological and autonomic symptoms, these phases and symptoms (mainly the psychiatric and neurological ones) can occur in a mixed form as in the case of our patient. Such an uncommon presentation of psychiatric and neurological symptoms before the autonomic instability should raise the clinician awareness to consider a diagnosis of an organic psychiatry disorder.

## Conclusions

Anti-NMDA receptor encephalitis is a serious and fatal disease if left untreated. However, there is no scientific support for anti-NMDA receptor encephalitis laboratory testing in patients with psychotic symptoms even though anti-NMDA receptor encephalitis presenting with psychosis is not a rare disease [10]. The hopeful goal of this case report is to raise clinician awareness of anti-NMDA receptor encephalitis in psychotic adolescent patients with atypical psychiatric presentation and concomitant neurological symptoms, such as speech alteration and motor changes, even in the absence of malignancies.

## Abbreviation

NMDA: *N*-methyl-d-aspartate
